# Supplementary material for: Dynamical origins of heat capacity changes in enzyme-catalysed reactions
Source: Nat Commun. 2018 Mar 21;9:1177. doi: 10.1038/s41467-018-03597-y (PMC5862990; doi:10.1038/s41467-018-03597-y)
Supplement: Supplementary file 1 — Supplementary Information(PDF 2383 kb) [file 41467_2018_3597_MOESM1_ESM.pdf]

Supplementary information

Dynamical origins of heat capacity changes in enzyme catalysed reactions

Marc W. van der Kamp[1,2†*], Erica J. Prentice[3†], Kirsty L. Kraakmann[3], Michael Connolly[2], Adrian J. Mulholland[2*] & Vickery L. Arcus[3*]

[1]School of Biochemistry, Biomedical Sciences Building, University Walk, University of Bristol, BS8 1TD, UK.
[2]Centre of Computational Chemistry, School of Chemistry, Cantock's Close, University of Bristol, BS8 1TS, UK.
[3]School of Science, University of Waikato, Hamilton, New Zealand

Email: marc.vanderkamp@bristol.ac.uk, adrian.mulholland@bristol.ac.uk, varcus@waikato.ac.nz

[†] These authors contributed equally to this work.

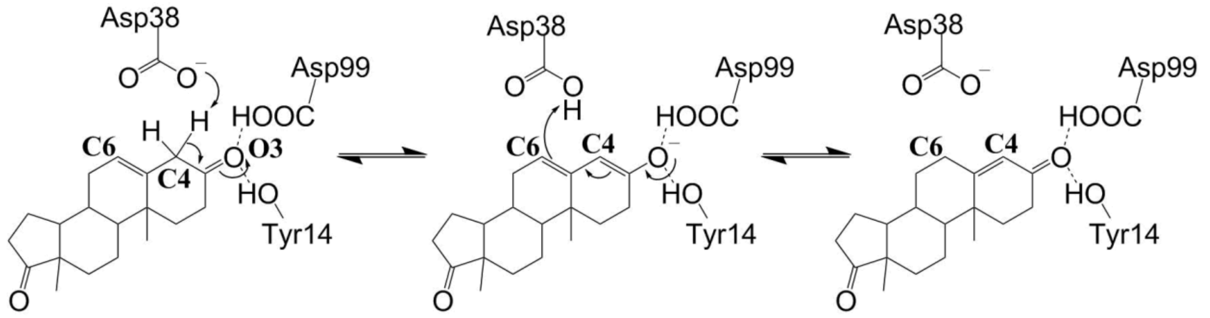

Supplementary Figure 1. Reaction mechanism of KSI. Conversion of 5–androstene-3,7–dione into 4–androstene-3,7–dione occurs through two consecutive proton transfers. The first proton transfer is from C4 to Asp38[1], and the second proton transfer is from Asp38 to C6[2]. In the intermediate state (used in the simulations as a proxy for the proton transfer transition states), Asp38 is protonated and hydrogen bonds donated by Tyr14 and Asp99 (indicated) stabilize the dienolate oxygen.

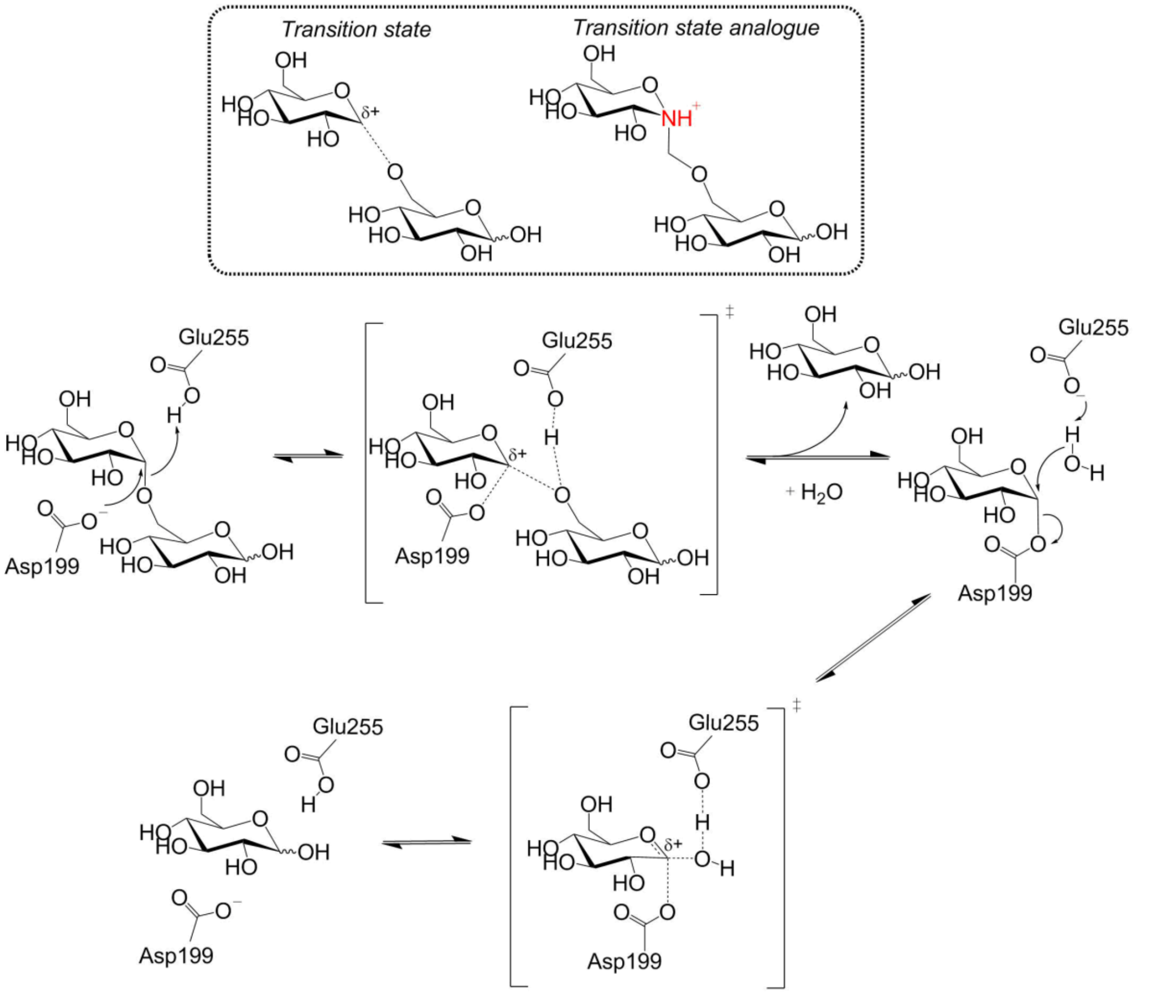

Supplementary Figure 2. Reaction mechanism of MalL[3] and TS analogue used in simulations. Cleavage of isomaltose (glc(α1-6)glc) into two D-glucose units catalysed by MalL proceeds via a two-step mechanism. In the first step, the +1 glucose unit is released, and a covalent glycosyl-enzyme intermediate is formed between Asp199 and the retained glucose unit. In the second step, the second glucose is released via nucleophilic attack by water. The retained glucose passes through a contorted half chair conformation as the anomeric carbon adopts a more planar conformation at the TS[4,5] (not shown). Top pane: transition state (TS) for the first MalL reaction step showing an elongated bond during the cleavage step and the oxocarbenium ion character. The charge and elongated bond are mimicked in the TS analogue via a charged nitrogen and methyl bridge, respectively.

Supplementary Note 1: Cloning, protein expression, purification, and characterization

Cloning, expression, purification and activity assays of MalL were as described previously[6]. Crystallization of MalL was performed using hanging drop vapor diffusion at 18 °C. Crystals were obtained by co-crystallization with 0.5 mM 1-deoxynojirimycin in 10 % Tacsimate™, pH 6.0 (Hampton Research, USA), 100 mM MES, pH 6.4, and 25 % polyethylene glycol 4,000. Crystals were flash cooled with cryoprotectant comprising of the crystallization mixture with 20 % glycerol for collection at 0.9537 Angstrom, 100 K, on the MX1 beamline at the Australian Synchrotron. Data was indexed and integrated in MOSFLM[7], and scaled in Scala[8]. Molecular replacement was performed with the WT MalL apo structure (PDB 4M56)[6] as the search model in Phenix MR[9]. Iterative rounds of refinement were performed in COOT and Phenix refine. 98 % of protein atoms are in the favoured Ramachandran regions, 2 % in the allowed regions, and none in the disallowed region.

The KSI sequence (*Pseudomonas testosteroni*) was optimized for expression in *Escherichia coli* and synthesized by GeneArt (Regensburg, Germany) for cloning into pET28B vector with a C-terminal hexa-His tag in *E. coli* BL21. Expression was carried out over ~24 hours in Luria-Bertani broth with 1 mM isopropyl β-D-1-thiogalactopyranoside at 28 °C. Purified KSI was obtained by a two-step immobilized metal affinity (IMAC)-gel filtration chromatography process. Cells were sonication lysed and loaded onto a HiTrap HP/FF column in buffer (50 mM TRIS, pH 7.0, 150 mM NaCl, 20 mM imidazole), and eluted over a linear gradient to high (1 M) imidazole over 50 ml. Protein containing fractions were pooled and further purified by gel filtration chromatography on a S200 10/300 analytical size exclusion column (GE Healthcare Life Science, UK) column equilibriated with 50 mM sodium phosphate, pH 7.0, 150 mM NaCl buffer. KSI activity was measured *in vitro* using a continuous enzyme assay following the isomerization of 19-nor-androst-5(10)-ene-3,17-dione at 248 nm. Assays were performed in 50 mM sodium phosphate, pH 7.0, with 150 mM NaCl for minimal pH change with temperature at a substrate concentration five times K_M.

Supplementary Note 2: Simulation protocols

For both KSI and MalL, the same simulation protocol was used. Histidine tautomers and Asn/His side-chain rotations of 180° were assigned according to the optimal hydrogen bond network[10]. For KSI, the following were rotated: Asn2, Asn104, His100 and His122 in chain A, and Asn19, Asn57 and Asn104 in chain B. No rotations were required for MalL. After addition of solvent (in tleap from AmberTools[11] with a closeness parameter of 0.9) and randomisation of the Na^+ ions (minimum distance between protein and Na^+ of 10 Å, minimum distance between Na^+ ions 5 Å), a minimisation procedure was started consisting of: 300 steps minimisation of water, ions and hydrogens only; 50 ps MD simulation in the NVT ensemble at 300 K of water and ions only (positional restraint on protein atoms: 25 $kcal \cdot mol^{-1} \cdot Å^{-2}$); minimisation of the whole system with positional restraints on Cα atoms (5 $kcal \cdot mol^{-1} \cdot Å^{-2}$) for 300 steps. Heating (random velocity assignment at 25 K followed by heating to 300 K for KSI and 320 K for MalL) was then performed in 20 ps simulation using Langevin dynamics for temperature control with a 1 ps^{-1} collision frequency (maintaining 5 $kcal \cdot mol^{-1} \cdot Å^{-2}$ positional restraints on Cα atoms). In four consecutive 10 ps simulations under the same conditions, the positional restraints on Cα atoms were reduced to 4, 3, 2, and 1 $kcal \cdot mol^{-1} \cdot Å^{-2}$. Subsequently, 1 ns of equilibration was performed in the NPT ensemble at 1 atm, using the Berendsen barostat (1 ps pressure relaxation time) and Langevin dynamics for temperature control (1 ps^{-1} collision frequency). Production simulation (500 ns) was then performed in the NVT ensemble with the Berendsen thermostat and loose temperature coupling (10 ps time constant), to limit the influence of the thermostat whilst avoiding temperature drift. In all dynamics simulations, the default direct-space cutoff was used for non-bonded interactions, with particle-mesh Ewald summation for long-range electrostatic interactions. Dynamics simulations were run with pmemd.cuda on GPUs, using the SPFP precision model[12].

Supplementary Note 3: Restraints in simulations

For KSI, restraints were required to keep Asp38 in a reactive (Michaelis-complex) conformation, in line with proton abstraction from C4 by Asp38 (Supplementary Figure 1). Without any restraints, the deprotonated Asp38 swings out into solvent within the initial 1ns NPT equilibration ($d\text{OD2}_{\text{Asp38}}$-$\text{C4}_{\text{substrate}}$ increased to 6.6-10.1; Supplementary Figure 3); once solvent exposed, the reactivity is severely limited[13]. Only after this initial change, instability of the loop that contains Asp38 occurs: the main-chain hydrogen bonds from Gly37 and Val40 (in the loop) to Ala114 are lost in the first 10 ns of simulation (donor-acceptor distances increase from <4 Å after NPT equilibration to >4.7 Å after 10 ns in all cases). The tendency of Asp38 to swing out into solvent may be a force-field limitation, or a consequence of the conformation of a deprotonated Asp38 directed to the C4-C5 double bond being a short-lived event prior to reaction (or a combination of both). To address whether the observed behaviour can be mitigated by using a more recent, improved protein force field, simulations of the reactant state (2 independent runs of 50 ns) without restraints were performed with Amber ff14SB. Essentially, the behaviour is the same as observed before (with ff99SB-ILDN): initial movement of Asp38 away from the substrate into solvent happens within the initial 1 ns NPT equilibration, and subsequently, the loop becomes less stable. Although the loop hydrogen bonds and structure may change less rapidly than with ff99SB-ILDN, hydrogen bonds still break within the first ~50 ns of simulation (Supplementary Figure 3d). This suggests that the movement of Asp38 into solvent followed by instability of the loop is not just an artefact of the ff99SB-ILDN force field used.

To keep Asp38 in a position relevant for the reactant state, a set of six restraints was used (Supplementary Figure 3). To allow reasonable sampling of the conformational fluctuations, 1) all restraints are one-sided (quasi-)harmonic, i.e. no restraint is applied if a distance stays within a certain range, 2) the restraint force-constant is mild, 25 $\text{kcal}\cdot\text{mol}^{-1}\cdot\text{Å}^{-2}$, and 3) all restraints are outside the range that is typically sampled in simulations of the IS state without restraints present. As a consequence, the simulations of IS with restraints in place that were used for analysis (for consistency), typically have no (or a very small) energy contribution from the restraints.

Despite the restraints imposed, Asp38 was still observed to move away from its Michaelis complex position in some simulations, i.e. the shortest possible proton transfer distance (*d*OH) moved beyond 4 Å and was consistently >4 Å for the remaining simulation time. Trajectories where this occurred were omitted from further analysis.

For MalL, initially no restraints were applied, but after many 10s of ns of simulation in some trajectories, the isomaltose moved out of the active site area. To avoid this from happening, a restraint was placed on the centre-of-mass distance between the heavy atoms of the −1 glucose unit on the one hand, and the Cα atoms of Asp199 and Asp332 on the other. This one-sided (quasi-)harmonic restraint is active when the distance is 10 Å or larger, with a force-constant of 15 $\text{kcal}\cdot\text{mol}^{-1}\cdot\text{Å}^{-2}$. It is only activated occasionally (<2% of the time), and stops the isomaltose substrate from drifting away.

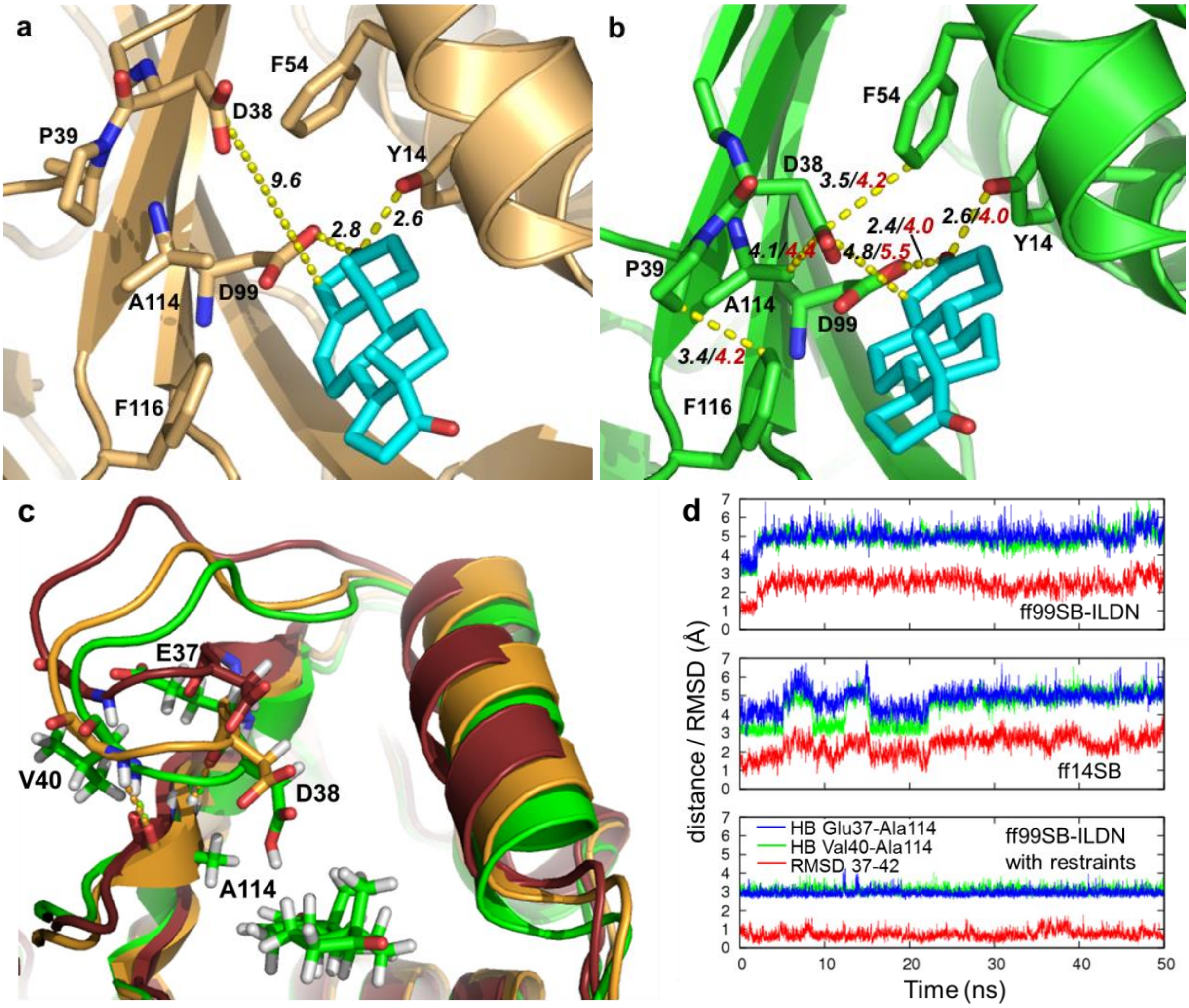

Supplementary Figure 3. Restraints required to maintain a Michaelis complex conformation in KSI simulations. a, Example snapshot from simulation of KSI with the reactant bound without restraints, indicating that Asp38 and the surrounding loop swing away from the bound substrate, into solvent. Distance between Asp38 Cγ and C4 on the substrate is indicated, alongside the hydrogen bond donor-acceptor distances between Tyr14, Asp99 and O3 (see Supplementary Figure 1). Hydrogens are omitted for clarity. **b**, Starting structure for simulation (with inhibitor bound, from PDB 1OHP) with distances on which restraints are placed to keep Asp38 in a reactive conformation indicated with dashed lines. Distances measured in the structure are shown in black, and distance where the one-sided harmonic restraint comes into effect in red. **c**, Example snapshots (ff99SB-ILDN, without restraints) indicating the sequential movement of Asp38 moving out into solvent (orange backbone, with the loop still anchored by backbone hydrogen bonds between Glu37-Ala114 and Val40-Ala114 – taken after 1 ns NPT equilibration), prior to further loop movement (dark red, taken after 10 ns of production simulation). Starting structure in green. **d**, Example distance plots in simulations with ff99SB-ILDN, ff14SB and ff99SB-ILDN with restraints. Change in loop structure (Cα RMSD of residues 37-42 after alignment on residues 1-116) and the presence/loss of backbone hydrogen bonds between Glu37-Ala114 and Val40-Ala114 (donor-acceptor distance) are indicated.

Supplementary Table 1. Crystallisation data for MalL complexed with 1-deoxynojirimycin.

**Data collection**		
Space group	P 2_1	
Cell dimensions:		
a,b,c (Å)	61.6,98.8,101.4	
α,β,γ (°)	90,103.8,90	
Resolution range (Å)	1.58-46.78	(1.58-1.67)
R_{merge}	0.078	(0.681)
$I / \sigma I$	16.4	(2.9)
Completeness (%)	98.8	(97.2)
Redundancy	9.3	(7.1)
**Refinement**		
No. of reflections	1,437,833	(146,565)
R_{work}/R_{free}	0.1698/0.1974	
No. of atoms		
Protein	9063	
Ligand	40	
Water	1267	
B-factors		
Protein	17.1	
Ligand	17.5	
Water	27.8	
RMSD		
Bond lengths (Å)	0.006	
Bond angles (°)	0.806	

Data was collected from one crystal. Values in brackets correspond to the outer resolution shell. The structure is available in the PDB under accession code 5WCZ.

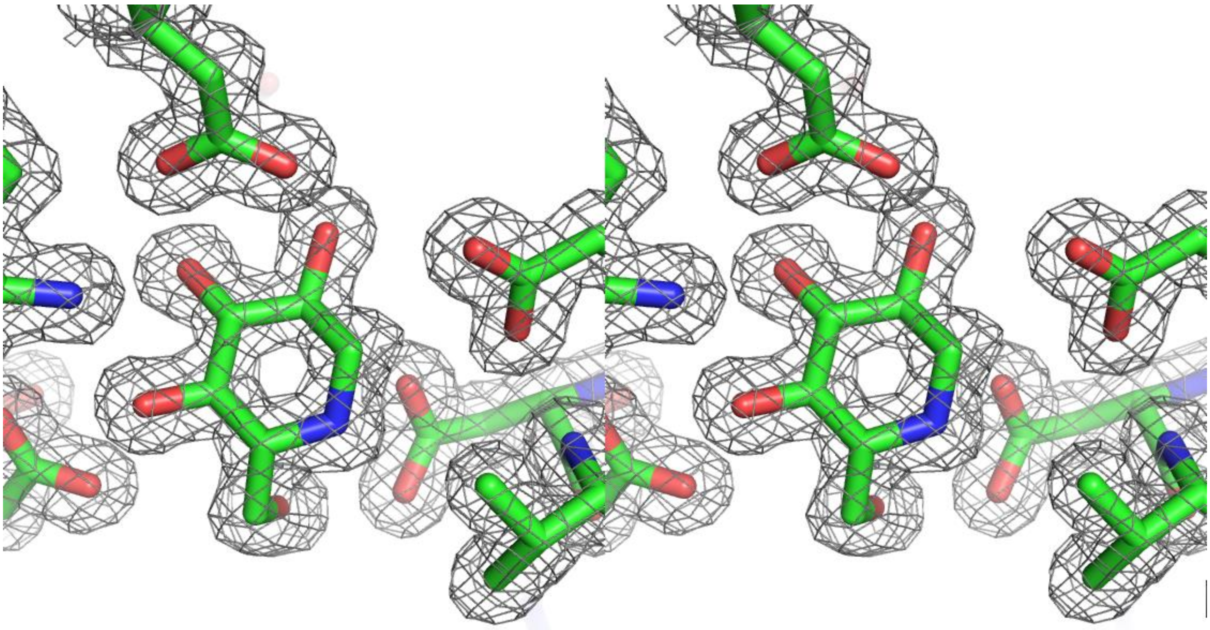

Supplementary Figure 4. 2Fo-Fc electron density map of 1-deoxynojirimycin and surrounding residues in the co-crystal structure with MalL (5WCZ). The density is contoured at 1 σ, and displayed in cross-eyed stereo-mode.

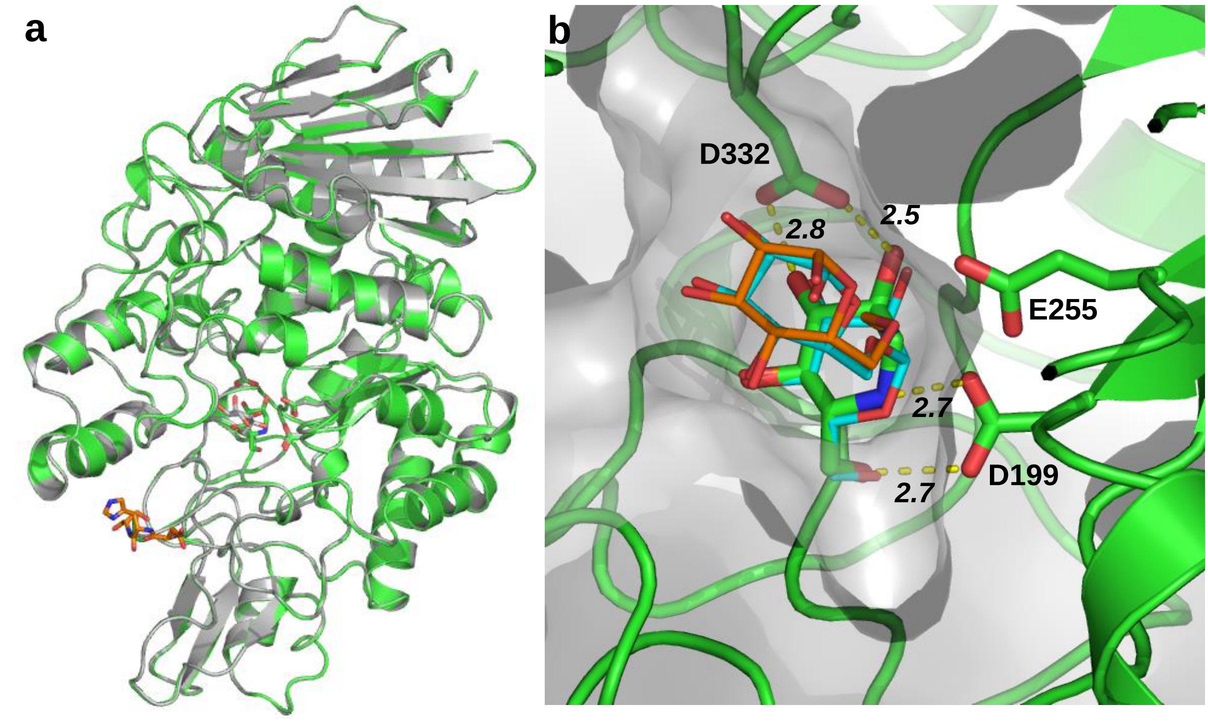

Supplementary Figure 5. Comparison of MalL structures with/without ligands bound. a, Structure of MalL complexed with 1-deoxynojirimycin (green carbons) compared to MalL with D-glucose in linear form in the active site (PDB 4M56; light gray). Missing residues 216-218 that were modelled in with COOT shown in sticks with orange carbons. **b**, Close-up of the active site of MalL complexed with 1-deoxynojirimycin (green carbons), with isomaltose (cyan carbons) and the transition state analogue (orange carbons) in their starting positions for simulation. Hydrogen-bond interactions between 1-deoxynojirimycin and MalL are indicated with dashed lines (and distances labeled in Å).

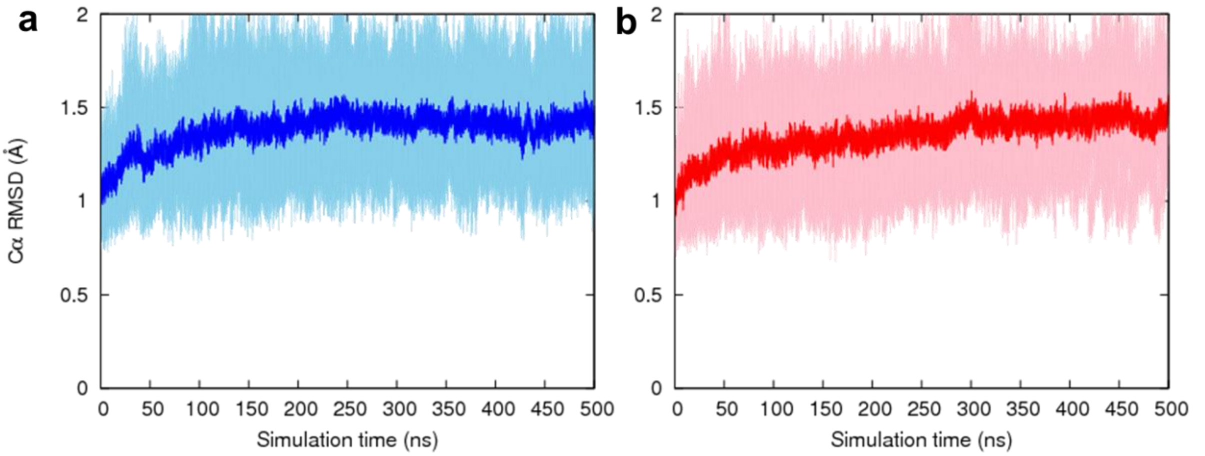

Supplementary Figure 6. Cα RMSD analysis of KSI simulations. a, RMSD for all ten reactant state trajectories (light blue), with the average for all 10 runs indicated by the blue line. **b**, RMSD for all ten intermediate state trajectories (pink), with the average for all 10 runs indicated by the red line. The plots indicate consistent conformational sampling across replicates and two states.

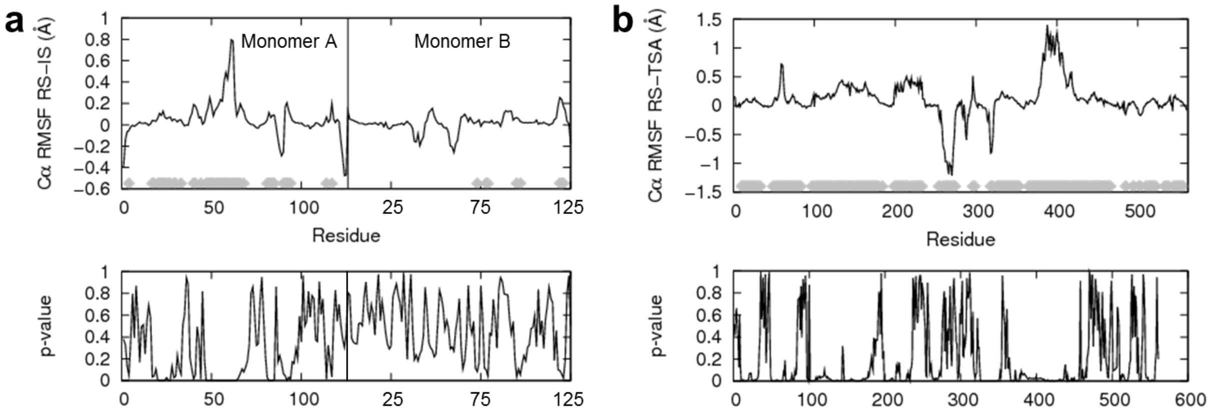

Supplementary Figure 7. Cα RMSF differences between states and their significance. a, Cα RMSF difference for KSI RS and IS states. **b**, Cα RMSF difference for MalL RS and TSA states. P-values at each residue are determined by a two-sample t-test between the two sets of Cα RMSF measurements and plotted below. P-values <0.01 are indicated in the top plot with gray diamonds.

Supplementary Note 4: Analysis of conformational sampling in MalL and outlier identification

During simulations of MalL (both states), Cα RMSD versus the starting structure rises significantly over the first 50 ns of simulation time in all trajectories (Supplementary Figure 7). From this point, individual trajectories trace a variety of RMSD trajectories; overall, simulations trend away further from the starting structure. Examination of individual runs shows an opening near the MalL active site as loops surrounding the active site move apart. This is observed in simulations of both the substrate and TS analogue bound state. Movement in loops 213-221, 387-417, and 287-302 away from the active site creates an 'opened' structure associated with larger RMSD (Supplementary Figure 7c-e).

Cluster analysis was performed on the MalL reactant and transition state analogue simulation sets to assess the conformational sampling across all runs, and to catch any simulations that significantly sample conformational space not observed in any of the other runs. The majority of replicate simulations cluster together; outliers from this represent more open conformations in latter simulation stages (Supplementary Figure 7c-f). As such are these are considered extremes on the closed-open continuum that is sampled in MalL simulations. The one exception to this is original run 8 with substrate bound, which is the sole occupant of a conformational cluster with 76% occupancy (Supplementary Table 2). Analysis of the trajectory indicates a significantly higher Cα RMSD than the other replicate runs, especially in the latter half of the simulation. Increases in RMSD from 120 ns onwards correspond to anomalous orientation of loops 288-300 and 380-420 (Supplementary Figure

8). Usually, the helix-loop-helix comprising of residues 380-420 sits into the active site; Glu389 in this region has been identified as significant for substrate recognition[14]. For a majority of the run, this interaction is broken as loop 288-300 flips into the active site, excluding helix-loop-helix 380-420. Based on the clustering results, high RMSD and the trapping of the structure in an anomalous loop configuration, this simulation was replaced with a new trajectory for the purposes of $\Delta C^{\ddagger}_{P}$ analysis.

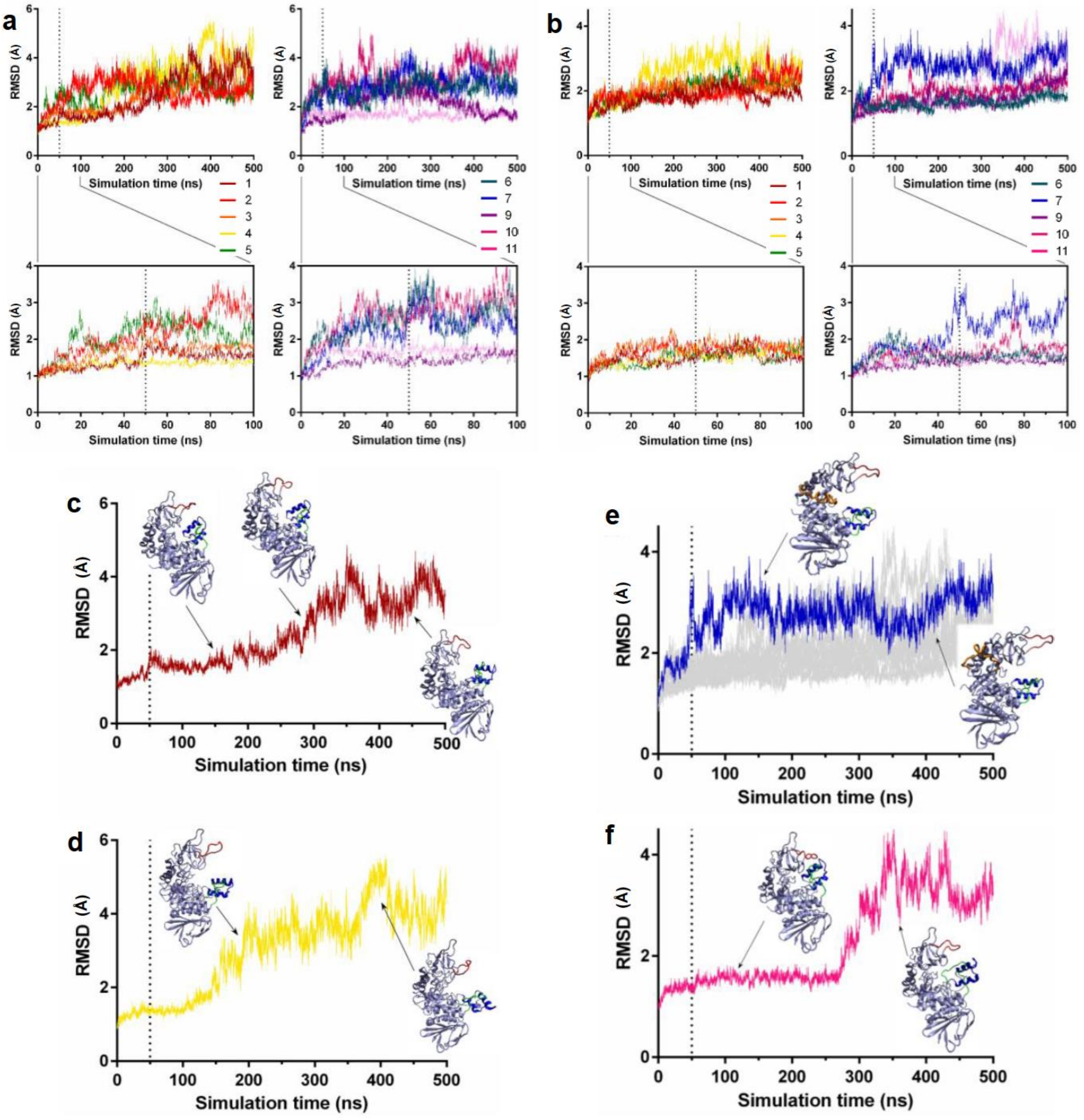

Supplementary Figure 8. Cα RMSD analysis of MalL simulations. a, Ten replica simulations of MalL with substrate bound. **b**, Ten replica simulations of MalL with transition state analogue bound. **c-d**, Example substrate bound simulations illustrating the active site opening. Loops contributing to the opening movement are indicated in red (residue 213-221), blue (387-417), and green (287-302). In **c**, gradual increases in RMSD up to 350 ns are accompanied by active site opening; in **c** and **d**, the largest changes occur due to the large opening of the helix-loop-helix (blue). **e-f**, Example transition state analogue bound simulations illustrating the active site opening. Loop colouring as in c-d. In **e**, RMSD plots of the remaining nine runs are traced as grey: the current run (blue line) has high RMSD compared to these due to early opening of loops about the active site and high mobility in loop 256-272 (orange). In **f**, a jump in RMSD is accompanied by an opening motion in loops about the active site, especially the helix-loop-helix motif (blue).

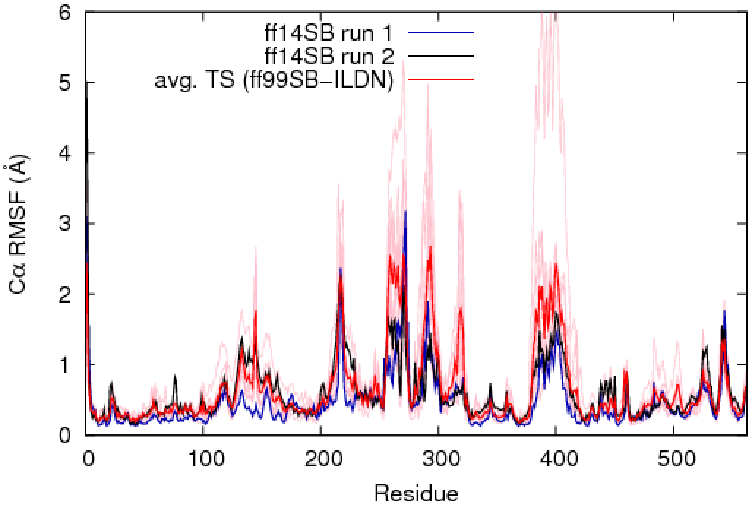

Supplementary Figure 9. Cα RMSF comparison of MalL simulations between force-fields. Cα RMSF for all ten transition state analogue simulations with ff99SB-ILDN are displayed as thin pink lines, with their average in red; two additional 500 ns simulations with ff14SB are shown as blue and black lines, respectively.

Supplementary Table 2. Cluster occupancies (fraction) in MalL simulation sets.

MalL RS	***Run***									
Cluster	**1**	**2**	**3**	**4**	**5**	**6**	**7**	**8**	**9**	**10**
1	0.604	0.967	0.535	0.312	0.413	0.34	0.931	0.044	0.973	0.081
2	0.361	0.033	0.455	0.276	0.587	0.446	0.069	0.14	0.024	0.535
3	0.001	0	7E-04	0	0	0	0	**0.756**	0	0.001
4	0.031	0	0.007	7E-04	0	0.034	0	0.053	0	0.345
5	0.004	0	0.002	0.411	0	0	0	0.007	0	0.033
6	0	0	4E-04	0	0	0.18	0	0	0.003	0.005
MalL RS new	**1**	**2**	**3**	**4**	**5**	**6**	**7**	**8**[a]	**9**	**10**
1	0.334	0.865	0.028	0.25	0.868	0.605	0.992	0	0.461	0.486
2	0.449	0.135	0.4	0.224	0.075	0.073	0.008	1.00	0.539	0.002
3	0.194	0	0.036	0.07	0.034	0.079	0	0	0	0.48
4	0.023	0	0.535	0.023	0.023	0.014	0	0	2E-04	0.003
5	0	0	0	0.432	0	7E-04	0	0	0	0.01
6	2E-04	0	4E-04	7E-04	2E-04	0.229	0	0	0	0.019
MalL TS	**1**	**2**	**3**	**4**	**5**	**6**	**7**	**8**	**9**	**10**
1	1.00	0.923	0.966	0.17	0.98	1.00	0.011	0.985	0.972	0.554
2	4E-04	0.046	0.034	0.005	7E-04	0	0.773	0.015	0.028	0
3	0	0.02	0	0.826	0.019	2E-04	0	0	0	0
4	0	0.01	0	0	0	0	0	0	0	0.446
5	0	2E-04	0	0	0	0	0.118	0	0	0
6	0	4E-04	0	0	4E-04	0	0.098	0	0	0

[a] Run 8 from the original set replaced (before clustering).

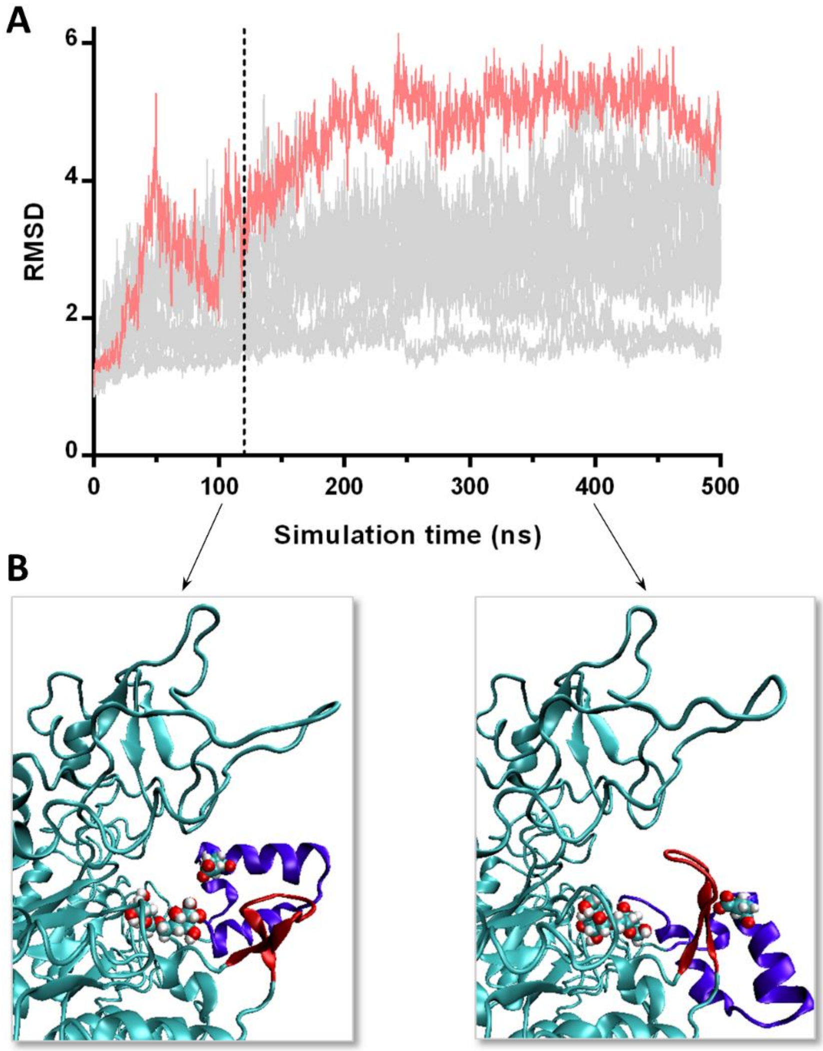

Supplementary Figure 10. Anomalous conformations sampled in a MalL reactant state simulation. **A**, RMSD plot of run 8 (pink) against all other runs (grey). The point where loop 288-300 first flips into the active site is indicated (vertical dashed line). **B**, Details of protein movements over the run. Loop 288-300 (red) flips into the active site channel around 120 ns, excluding helix-loop-helix 380-420 (blue) from the normal orientation (left). The substrate and substrate interacting residue Glu389[14] are shown as spheres.

Supplementary Note 5: Conformational clustering

For KSI, all conformations sampled were divided into two separate conformational clusters for both simulated states. This was done on the basis that for both states, only two highly similar significant clusters are present (Figure 2 and Supplementary Table 4). To ensure all sampled conformations are included in the analysis, the K-means algorithm was used (see Methods). Because in this case, substantially different conformational clusters exist (with substantially different variances; Figure 2), it is relevant to analyse these clusters separately: reactions take place very rapidly if the energy barrier to reaction is overcome (e.g. proton transfer on the order of ~fs) and a transition state is (by definition) very short-lived. The enzyme conformation will thus not change from one cluster to another on this timescale. From experiment, $\Delta C_P^{\ddagger}$ is inferred from steady-state kinetics and its dependence on temperature, involving a very large number of individual enzyme molecules. If the simulations are sufficiently accurate, clustering will identify different relevant conformations (and their fractions) in the overall population of enzymes present in experiment.

Combined clustering of the 20 MalL simulations (10 for each state) using the hierarchical agglomerative algorithm with a minimum cluster distance (epsilon) of 2.1 Å leads to 9 clusters with an overall contribution >1%. Only the top two clusters are sampled significantly in both states (of the remaining clusters, one has a 0.58% contribution of the minor contributing state; all others are 0.08% or less).

Supplementary Table 3. Hierarchical agglomerative clustering of KSI simulations with different minimum distances between clusters (ε).

	RS		IS	
ε	cluster ID	cluster fraction[a]	cluster ID	cluster fraction[a]
1.5	**0**	**0.482**	**0**	**0.589**
	**1**	**0.365**	**1**	**0.318**
	2	0.033	2	0.036
	3	0.033	3	0.022
	4	0.017	4	0.015
	5	0.016	5	0.008
	6	0.014	6	0.007
	7	0.014	7	0.003
	8	0.013	8	0.002
	9	0.010	9	0.001
	10	0.001		
	11	0.001		
	12	0.001		
	13	0.000		
	14	0.000		
1.6	**0**	**0.498**	**0**	**0.613**
	**1**	**0.465**	**1**	**0.383**
	2	0.018	2	0.003
	3	0.011	3	0.002
	4	0.005		
	5	0.002		
	6	0.001		
1.7	**0**	**0.509**	**0**	**0.993**
	**1**	**0.485**	1	0.004
	2	0.005	2	0.003
	3	0.002		
1.8	**0**	**0.995**	**0**	**0.996**
	1	0.005	1	0.004

[a] Clusters with fractions >4% are shown in bold.

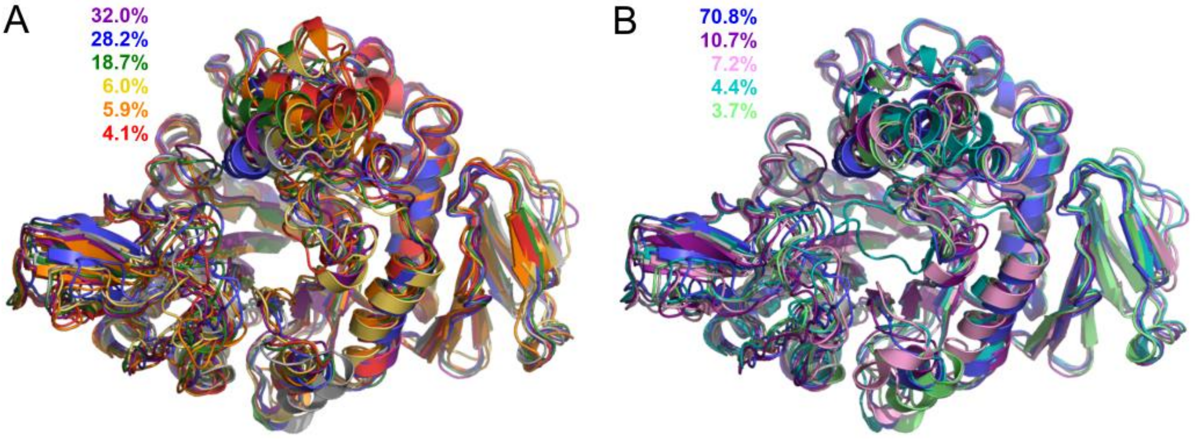

Supplementary Figure 11. Clustering of MalL simulations. A, Representative structures of the 6 clusters with >2% occupation in the reactant state simulations. **B**, Representative structures of the 5 clusters with >2% occupation in the transition state analogue simulations.

Supplementary Table 4. Average variances (kJ·mol^{-1}) in MalL simulations with different moving average windows and calculated $\Delta C_P^{\ddagger}$ values (kJ·mol^{-1}·K^{-1}).

Window (ns) / Run	*5*	*10*	*20*	*30*	*40*	*50*	*60*	*70*	*80*
RS1	98934	107952	117877	123985	128858	133199	137578	141595	145424
RS2	97766	109106	118811	122010	123875	124766	125580	126818	127813
RS3	102634	112815	121874	125884	129107	131912	134314	136289	137796
RS4	100083	108057	116534	122401	126454	129299	131138	132989	134795
RS5	96523	105506	115887	123096	128154	131739	134025	135976	137667
RS6	97118	105787	114536	119363	123393	127081	130702	134300	137632
RS7	101626	112744	121029	124207	126018	127573	128375	129033	129753
RS8	95025	105083	114226	119188	122202	124123	125594	126835	128156
RS9	102249	115060	130063	138746	144370	148900	151995	153992	155579
RS10	100899	111640	119972	124923	128023	130081	131836	133613	135434
TS1	100744	111520	119423	122700	125002	126384	127710	128972	130215
TS2	96771	105173	112134	115513	118088	119613	121291	123177	125123
TS3	95271	104321	109936	112497	114033	114832	115362	115792	116011
TS4	100410	110043	120530	127065	132570	137922	143160	148017	152907
TS5	101713	110658	119383	123880	125998	126723	127886	129424	130750
TS6	90113	97911	107421	113356	117254	120333	123344	126312	128651
TS7	106431	120394	134759	143165	151196	157813	162873	165730	167310
TS8	93609	102385	110629	113835	115501	117084	118159	118640	119538
TS9	98765	108197	117117	122829	126604	129588	132198	134276	135931
TS10	101408	113701	125321	130424	134154	137136	139135	140811	142248
Average RS	99286	109375	119081	124380	128045	130867	133114	135144	137005
Std. Dev.	2617	3508	4660	5502	6219	7019	7644	8043	8433
Average TS	98524	108430	117665	122526	126040	128743	131112	133115	134868
Std. Dev.	4692	6349	8234	9547	11247	12883	14197	15025	15622
RS-TS Difference	-762	-945	-1416	-1854	-2006	-2124	-2002	-2029	-2136
$\Delta C^{\ddagger}_{P}$	**-3.75**	**-4.64**	**-6.96**	**-9.11**	**-9.86**	**-10.44**	**-9.84**	**-9.97**	**-10.50**
Std. Dev.	0.28	0.42	0.76	1.11	1.36	1.60	1.63	1.72	1.86

Supplementary Note 6: Calculation of variances and $\Delta C_P^{\ddagger}$ with solvent

The contribution of solvent to the variance of the force-field energies for the two states (from which $\Delta C_P^{\ddagger}$ is calculated) may be significant, e.g. due to electrostatic screening that solvent would provide. In an attempt to include possible solvent effects, we originally intended to calculate $\Delta C_P^{\ddagger}$ values based on variances of energies obtained with a layer of explicit solvent (approximately the 1st and 2nd solvation shell) around the protein structures. However, there is no clear way to select which water m olecules should be included in such a calculation, and errors in the energies of water modelled by force-field approaches (see e.g. [15]) may be a further source of error for the resulting heat capacities. We therefore used a Poisson-Boltzmann implicit solvent model (as implemented in Amber), see Supplementary Table 4. The resulting $\Delta C_P^{\ddagger}$ value for MalL converges less well with moving variance window size, but for both enzymes the values are qualitatively similar to those obtained without solvent. We can

thus conclude that no large error is introduced by excluding contributions of solvent in calculation of the *difference* in variance between two states, from which $\Delta C_P^{\ddagger}$ is calculated.

Supplementary Table 5. Calculated $\Delta C_P^{\ddagger}$ values (kJ·mol^{-1}·K^{-1}) and their standard deviations for different subsections of the two enzymes.

Ketosteroid isomerase (KSI)			***MalL***		
**Region**	$\Delta C_P^{\ddagger}$	**Std. Dev.**[a]	**Region**	$\Delta C_P^{\ddagger}$	**Std. Dev.**[b]
Total (A+B)*	−0.63	0.34	Total (1−561*)	−9.97	1.72
Backbone[c]	−0.21	0.04	Backbone[c]	−7.57	0.61
Monomer A*	1.77	1.65	1−193	−7.02	0.88
Monomer B	−1.20	0.59	194−373*	6.92	1.84
1-45 (A)	1.96	2.62	374−459	−20.76	5.11
46-70 (A)	−1.80	2.04	460−561	1.31	0.13
71-125* (A)	1.15	1.23	194−249	1.36	0.30
			250−321*	7.29	2.50
			322−373	−1.08	0.11
Total* + PBSA[e]	−2.26	0.63	Total* + PBSA[e]	−3.84[d]	0.19

* indicates that the ligand is included. [a]Standard deviation calculated as the sum of the standard deviations of RS and IS variances computed using a leave-one-out procedure (one simulation of each state is omitted at a time). [b]Standard deviation calculated from the sum of the standard deviations of RS and TSA moving variances for each of the 10 simulations. [c]Variances obtained from force-field energies calculated after deleting all amino-acid side-chains. [d]For consistency, the moving variance window was again 70 ns (in line with the calculations without implicit solvent), but $\Delta C_P^{\ddagger}$ does not converge well with window size (with 60 or 80 ns windows, $\Delta C_P^{\ddagger}$ is −3.54 and −4.06 kJ·mol^{-1}·K^{-1}, respectively). [e]PBSA energy calculations were performed with sander from AmberTools 16. Total electrostatic energy and forces were calculated with the particle-particle-particle mesh (P3M) procedure, and the cut-off distance for van der Waals interactions was 8 Å. The 'mbondi' set of atomic radii was used.

Supplementary References

1 Pollack, R. M., Thornburg, L. D., Wu, Z. R. & Summers, M. F. Mechanistic insights from the three-dimensional structure of 3-oxo-Delta(5)-steroid isomerase. *Arch. Biochem. Biophys.* **370**, 9-15 (1999).

2 Kraut, D. A. *et al.* Testing electrostatic complementarity in enzyme catalysis: Hydrogen bonding in the ketosteroid isomerase oxyanion hole. *Plos Biol* **4**, 501-519 (2006).

3 Zechel, D. L. & Withers, S. G. Glycosidase mechanisms: Anatomy of a finely tuned catalyst. *Accounts of Chemical Research* **33**, 11-18, doi:10.1021/ar970172+ (2000).

4 Rye, C. S. & Withers, S. G. Glycosidase mechanisms. *Current Opinion in Chemical Biology* **4**, 573-580, doi: 10.1016/S1367-5931(00)00135-6 (2000).

5 Vasella, A., Davies, G. J. & Böhm, M. Glycosidase mechanisms. *Current Opinion in Chemical Biology* **6**, 619-629, doi: 10.1016/S1367-5931(02)00380-0 (2002).

6 Hobbs, J. K. *et al.* Change in heat capacity for enzyme catalysis determines temperature dependence of enzyme catalyzed rates. *ACS Chemical Biology* **8**, 2388–2393, doi:10.1021/cb4005029 (2013).

7 Leslie, A. G. The integration of macromolecular diffraction data. *Acta Crystallogr D Biol Crystallogr* **62**, 48-57, doi:10.1107/S0907444905039107 (2006).

8 Evans, P. Scaling and assessment of data quality. *Acta Crystallogr D Biol Crystallogr* **62**, 72-82, doi:10.1107/S0907444905036693 (2006).

9 Adams, P. D. *et al.* PHENIX: a comprehensive Python-based system for macromolecular structure solution. *Acta Crystallogr D Biol Crystallogr* **66**, 213-221, doi:10.1107/S0907444909052925 (2010).

10 Hooft, R. W. W., Sander, C. & Vriend, G. Positioning hydrogen atoms by optimizing hydrogen-bond networks in protein structures. *Proteins* **26**, 363-376 (1996).

11 D.A. Case, D. S. C., T.E. Cheatham, III, T.A. Darden, R.E. Duke, T.J. Giese, H. Gohlke, A.W. Goetz, D. Greene, N. Homeyer, S. Izadi, A. Kovalenko, T.S. Lee, S. LeGrand, P. Li, C. Lin, J. Liu, T. Luchko, R. Luo, D. Mermelstein, K.M. Merz, G. Monard, H. Nguyen, I. Omelyan, A. Onufriev, F. Pan, R. Qi, D.R. Roe, A. Roitberg, C. Sagui, C.L. Simmerling, W.M. Botello-Smith, J. Swails, R.C. Walker, J. Wang, R.M. Wolf, X. Wu, L. Xiao, D.M. York, P.A. Kollman *AMBER 2014* (University of California, San Francisco.).

12 Salomon-Ferrer, R., Gotz, A. W., Poole, D., Le Grand, S. & Walker, R. C. Routine microsecond molecular dynamics simulations with AMBER on GPUs. 2. Explicit solvent particle mesh Ewald. *J Chem Theory Comput* **9**, 3878-3888, doi:10.1021/ct400314y (2013).

13 Van der Kamp, M. W., Chaudret, R. & Mulholland, A. J. QM/MM modelling of ketosteroid isomerase reactivity indicates that active site closure is integral to catalysis. *FEBS J.* **280**, 3120-3131 (2013).

14 Yamamoto, K., Miyake, H., Kusunoki, M. & Osaki, S. Steric hindrance by 2 amino acid residues determines the substrate specificity of isomaltase from *Saccharomyces cerevisiae*. *Journal of Bioscience and Bioengineering* **112**, 545-550 (2011).

15 Levitt, M., Hirshberg, M., Sharon, R., Laidig, K. E. & Daggett, V. Calibration and testing of a water model for simulation of the molecular dynamics of proteins and nucleic acids in solution. *J Phys Chem B* **101**, 5051-5061 (1997).
